# Long-term live-cell imaging of GFAP+ astroglia and laminin+ vessels in organotypic mouse brain slices using microcontact printing

**DOI:** 10.3389/fncel.2025.1540150

**Published:** 2025-01-27

**Authors:** Christian Humpel

**Affiliations:** Laboratory of Psychiatry and Experimental Alzheimer’s Research, Medical University of Innsbruck, Innsbruck, Austria

**Keywords:** organotypic brain slices, microcontact printing, collagen, live-cell imaging, GFAP, laminin

## Abstract

Organotypic brain slices are three-dimensional, 150-μm-thick sections derived from postnatal day 10 mice that can be cultured for several weeks *in vitro*. However, these slices pose challenges for live-cell imaging due to their thickness, particularly without access to expensive two-photon microscopy. In this study, we present an innovative method to label and visualize specific brain cell populations in living slices. Using microcontact printing, antibodies are applied directly onto the slices in a controlled 400-μm-diameter pattern. Astrocytes are labeled with glial fibrillary acidic protein (GFAP), and vessels are labeled with laminin. Subsequently, slices are incubated with secondary fluorescent antibodies (green fluorescent Alexa-488 or red fluorescent Alexa-546) and visualized using an inverted fluorescence microscope. This approach offers a cost-effective and detailed visualization technique for astroglia and vessels in living brain slices, enabling investigation to be conducted over several weeks.

## Introduction

### Organotypic brain slices

Organotypic brain slice cultures provide a cytoarchitecturally intact, three-dimensional brain model that can be studied *ex vivo* (Gähwiler, 1981; Stoppini et al., 1991; Humpel, 2015). These cultures enable the simultaneous investigation of multiple variables, reducing experimental variability and bridging the manipulability of *in vitro* models with the physiological integrity of *in vivo* models. Furthermore, this model significantly reduces reliance on animal experiments as multiple slices can be derived from a single brain. While acute brain slices are typically used for electrophysiological studies, chronic setups allow many brain regions to be cultured for weeks. Notably, organotypic brain slice cultures also facilitate co-culture experiments, enabling the interaction of two or more connected brain areas to be studied. Typically, these slices are cultured on semipermeable 0.4-μm pore inserts positioned between a humidified atmosphere and the culture medium, allowing them to attach to the membrane and absorb nutrients through the porous surface (detailed review Humpel, 2015 and methodological review Humpel, 2018).

### Collagen hydrogel-based microcontact prints

Microcontact printing (μCP) is an effective, cost-efficient method for patterning biomolecules on various substrates with high resolution (sub-μm range). This technique offers a powerful tool for engineering cellular behavior by microprinting proteins of interest into defined patterns. Our laboratory developed a collagen hydrogel-based approach that enables nearly all biomolecules to be microcontact printed (review Steiner and Humpel, 2023). The hydrogel can be applied directly to a brain region, gradually releasing the biomolecule to the organotypic brain slice, facilitating precise localization and controlled delivery.

Alternatively, collagen scaffolds printed near brain slices facilitated the study of cell outgrowth, marking the first integration of microcontact printing with organotypic brain slices (review Steiner and Humpel, 2023). First, we could investigate nerve fiber growth along such microcontact prints; for example, cholinergic neurons grew along nerve growth factor (NGF)-printed patterns (Steiner and Humpel, 2021). Second, we embedded monocyte chemoattractant protein-1 (MCP-1) into the collagen hydrogel prints, connecting them with cortical brain slices (Steiner and Humpel, 2022), and observed strong Iba1-positive microglial migration originating from the slices (Steiner and Humpel, 2022). Third, endothelial cells were activated and migrated along the microcontact prints, being able to form new vessels (Steiner and Humpel, 2024a,b). Finally, we recently showed that μCP-printed human plasma from Alzheimer’s disease (AD) patients decreased the migration of Iba1+ microglia along the plasma, allowing the identification of novel biomarkers (Steiner et al., 2024).

### Live-cell imaging of brain slices

Microscopic examination of cells within thick organotypic brain slices presents significant challenges. While two-photon microscopy allows deep tissue imaging (Dürst and Oertner, 2022; Pappinen et al., 2012), it is expensive and primarily available in select institutions. Current techniques often rely on paraformaldehyde-fixed tissue sections, where antibodies do not penetrate deep into the tissue. Although our postnatal brain slices are 150 μm thick, imaging is typically limited to the slice surface. Moreover, live-cell imaging is constrained by the inability of dyes to penetrate deeply or by their cytotoxicity. A PubMed search with the keywords (organotypic [Title]) AND (live imaging) AND (brain) revealed only 17 relevant articles (November 2024).

Live-cell imaging in organotypic brain slices is challenging due to the technical limitations of thick tissue sections. One approach involves using adeno-associated viruses, which enable live-cell imaging but require specialized safety laboratories (Kennedy and Rinholm, 2017). Another common method is utilizing transgenic mice expressing reporter genes, such as green fluorescent protein (GFP). These mice have been used effectively in several studies (Gogolla et al., 2006; Llufriu-Dabén et al., 2019; Brachmann and Tucker, 2011), as well as in our laboratory (Daschil and Humpel, 2016). However, maintaining transgenic mouse lines can be prohibitively expensive, limiting broader applicability.

Other techniques include *in utero* electroporation (Eid et al., 2018) and lipid-based transfection methods (Gogolla et al., 2006), which offer alternatives but are constrained by low efficiency and the need for a gene of interest. Calcium imaging (Pozzo Miller et al., 1993; Tominaga et al., 1994), which uses fluorescent dyes such as Rhod-4 (red) or Fluo-4 (green), is another established technique that has been widely used in neurobiology for decades (Pozzo Miller et al., 1993; Tominaga et al., 1994). We have also validated calcium imaging in our laboratory, with successful application to organotypic slices (Steiner and Humpel, 2024a,b). However, calcium dyes are limited by shallow tissue penetration and high background fluorescence upon activation.

The gene gun method, which uses ballistic delivery of DNA vectors via gold particles, is a promising alternative for live-cell imaging applications (Benediktsson et al., 2005). Despite its potential, this approach is hindered by the high cost of gold particles and the need for gene plasmids and specialized equipment. These methods highlight the difficulties of live-cell imaging in organotypic brain slices, further compounded by the high costs and complexity of existing techniques.

These challenges underscore the need for novel and accessible methods to enable efficient protein labeling and long-term imaging of cellular dynamics in brain slices, addressing limitations in current approaches.

This study presents a novel method for fluorescently labeling astroglia (using GFAP antibodies) and vessels (using laminin antibodies) in organotypic brain slices with high spatial precision, achieved through microcontact printing in 400-μm-diameter patterns. We provide a detailed description of this technique, demonstrate its application in live-cell imaging, and discuss its limitations and potential future perspectives.

## Materials and methods

### Organotypic brain slices

**Figure 1:**
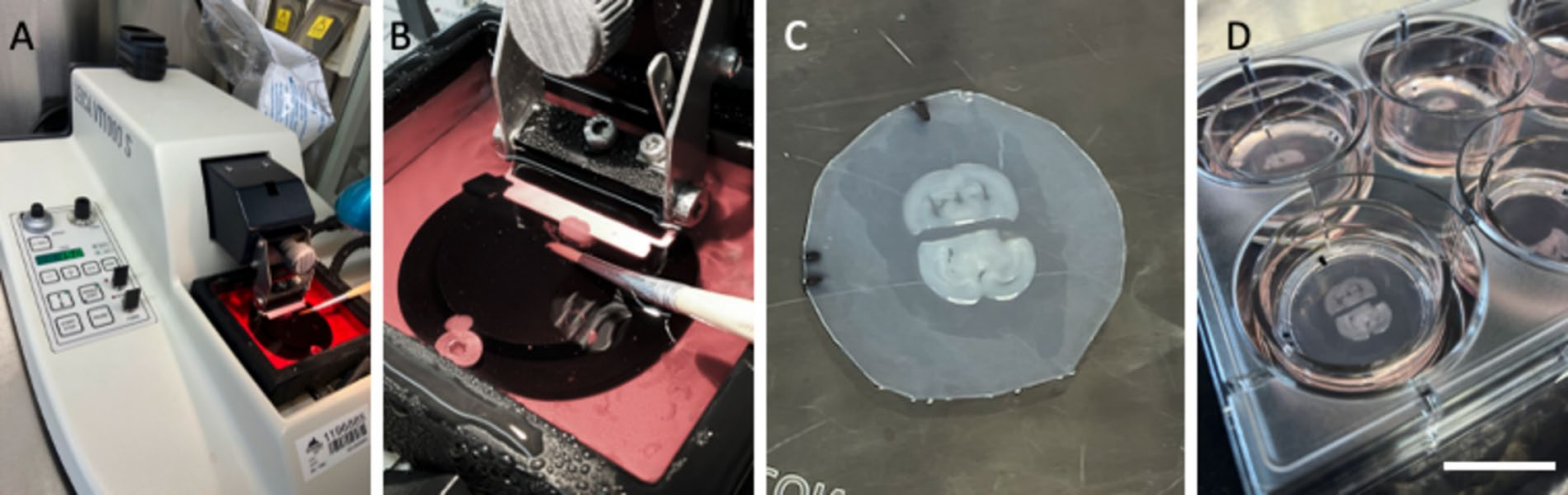
Setup of organotypic brain slices from postnatal mice. Brains from postnatal day 8–10 mice were sectioned into 150-μm-thick slices using a Leica V1000S vibratome **(A)**, and whole-brain sections were collected in culture media **(B)**. Each brain section was halved, and two half-brain slices from two different mice (*n* = 2) were placed on a semipermeable membrane (Biopore BGCM0010) with a space of ~2 mm **(C)**. These membranes and the brain slices were cultured on 0.4-μm membrane inserts (PICM03050) in 6-well plates at 37°C and 5% CO_2_ **(D)**. The black lines in **(C)** allow us to mark the alignment and orientation of the slices, the space, and the location of the prints. Scale bars: A = 12 cm, B = 1.8 cm, C = 0.9 cm, D = 2.5 cm.

The preparation of organotypic brain slices has been extensively documented in previous reviews (Humpel, 2015, 2018). Briefly, 150-μm-thick brain slices are prepared from postnatal day 8–10 C57BL6 mice using a vibratome (Leica, VT1000S) (Figures 1A,B) under cooled conditions (~5°C, Julabo F250 cooling system). For this study, half-brain slices at the hippocampal level were obtained (Figure 1C). They were placed approximately 2 mm apart on Biopore membranes (10FT roll, BGCM0010, Merck Millipore) and subsequently mounted onto 0.4-μm membrane inserts (Merck Millipore, PICM03050). To mark the alignment and orientation of the slices, the membranes are marked with a small black fine liner (Figure 1C). The slices were cultured (Figure 1D) in 6-well plates (Sarstedt, 83.3920) within a CO_2_ incubator at 37°C (5%) for 2–3 weeks in slice medium containing minimal essential medium (16.1 g/L; MEM, Gibco, 11012044), supplemented with NaHCO₃ (0.43 g/L), glucose (6.25 g/L; Merck, 8342), glutamine (116 mg/L; Merck, 1.00289.0100), 10% horse serum (Gibco, 16050-122, Lot: 2320064), 25% Hanks’ Balanced Salt Solution (HBSS, Gibco, 24020091), and 1× antibiotic-antimycotic solution (Gibco, 15240-062), adjusted to a pH of 7.2. The medium was replaced weekly.

All experiments were approved by the Austrian Ministry of Science and Research, complied with Austrian animal welfare guidelines, and adhered to the principles of the 3Rs (replace, reduce, and refine).

### Preparation of stamps for microcontact printing

**Figure 2:**
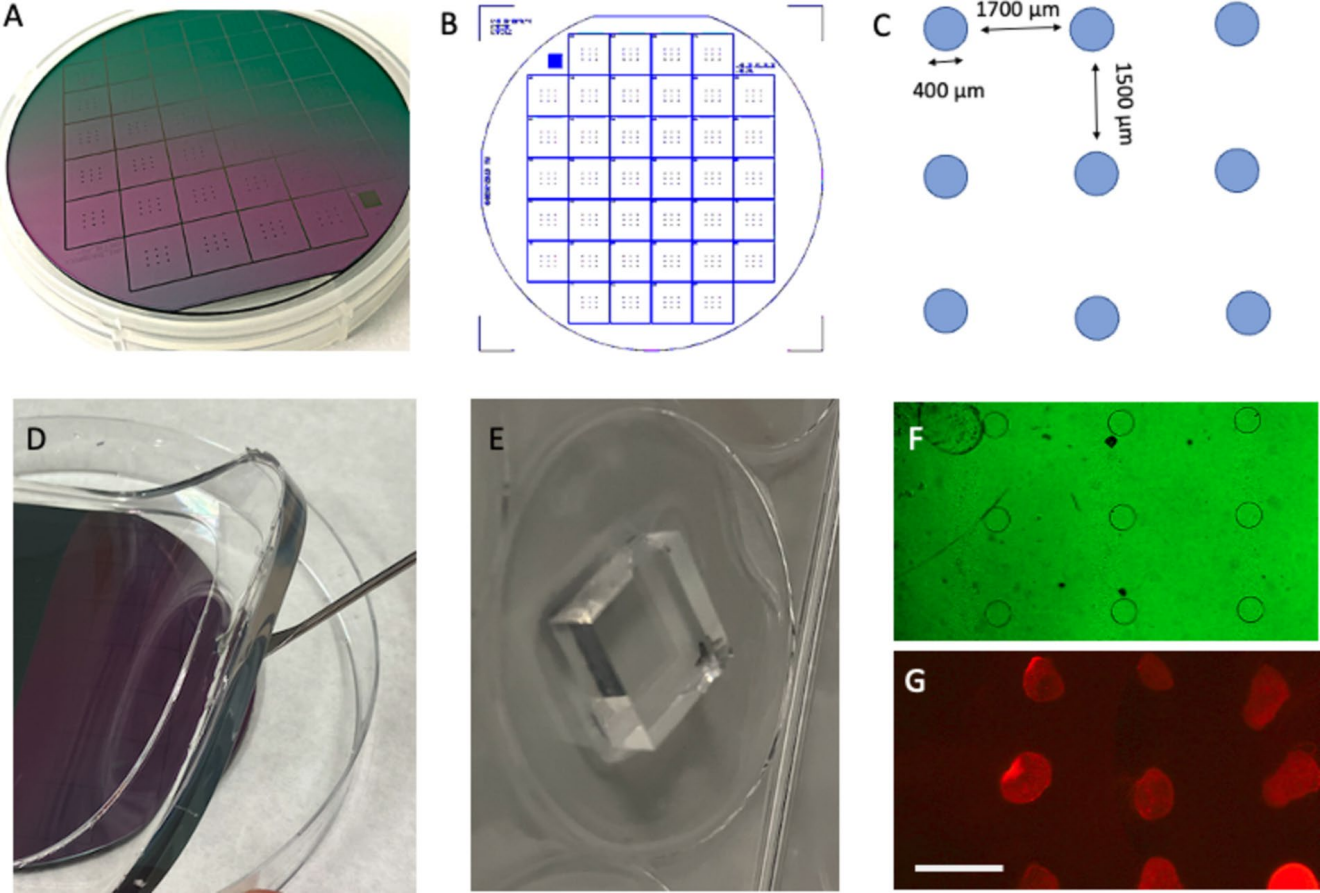
Preparation of stamps from a master plate. A master plate with a diameter of 10 cm **(A)** was constructed by GeSiM (Germany) and contains 38 stamps **(B)**. Each stamp has nine spots with a diameter of 400 μm, with a horizontal space of 1700 μm and vertical spacing of 1,500 μm **(C)**. The stamps are made from polydimethylsiloxane (PDMS) using the master plate as a mold **(D)**, and each stamp is cut out using a scalpel **(E)**. Phase contrast microscopy revealed the nine dots **(F)**, which are fluorescently labeled using a red fluorescent Alexa-546 antibody printed on an extra membrane **(G)**. Scale bars: A = 2.2 cm, B = 2.5 cm, E = 6.7 mm, F = 1,250 μm, and G = 1700 μm.

Polydimethylsiloxane (PDMS) stamps were prepared using silicon wafer templates (“master” molds). Each master mold (3×3 with 400-μm-diameter dots) was purchased from GeSiM (Ges. für Silizium-Mikrosysteme, Großerkmannsdorf, Germany), enabling the production of 38 stamps (Figures 2A–C).

- Form PDMS stamp’s surface relief by casting and curing liquid prepolymer PDMS against the silicon wafer master mold.
- Mix the elastomer base solution and curing agent in a 1:10 ratio using gentle stirring.
- Place the silicon master mold in the center of a Petri dish and pour the liquid PDMS over it.
- Remove air bubbles with a desiccator connected to a vacuum pump.
- Cure the PDMS at 60°C overnight.
- Peel off the cured PDMS from the silicon master mold and cut the stamps to the desired size using a scalpel (Figures 2D,E).
- Sterilize the stamps under UV light for 10 min.
- Check the dots under the microscope after labeling (Figures 2F,G).

### Preparation of the collagen-loaded stamps

**Figure 3:**
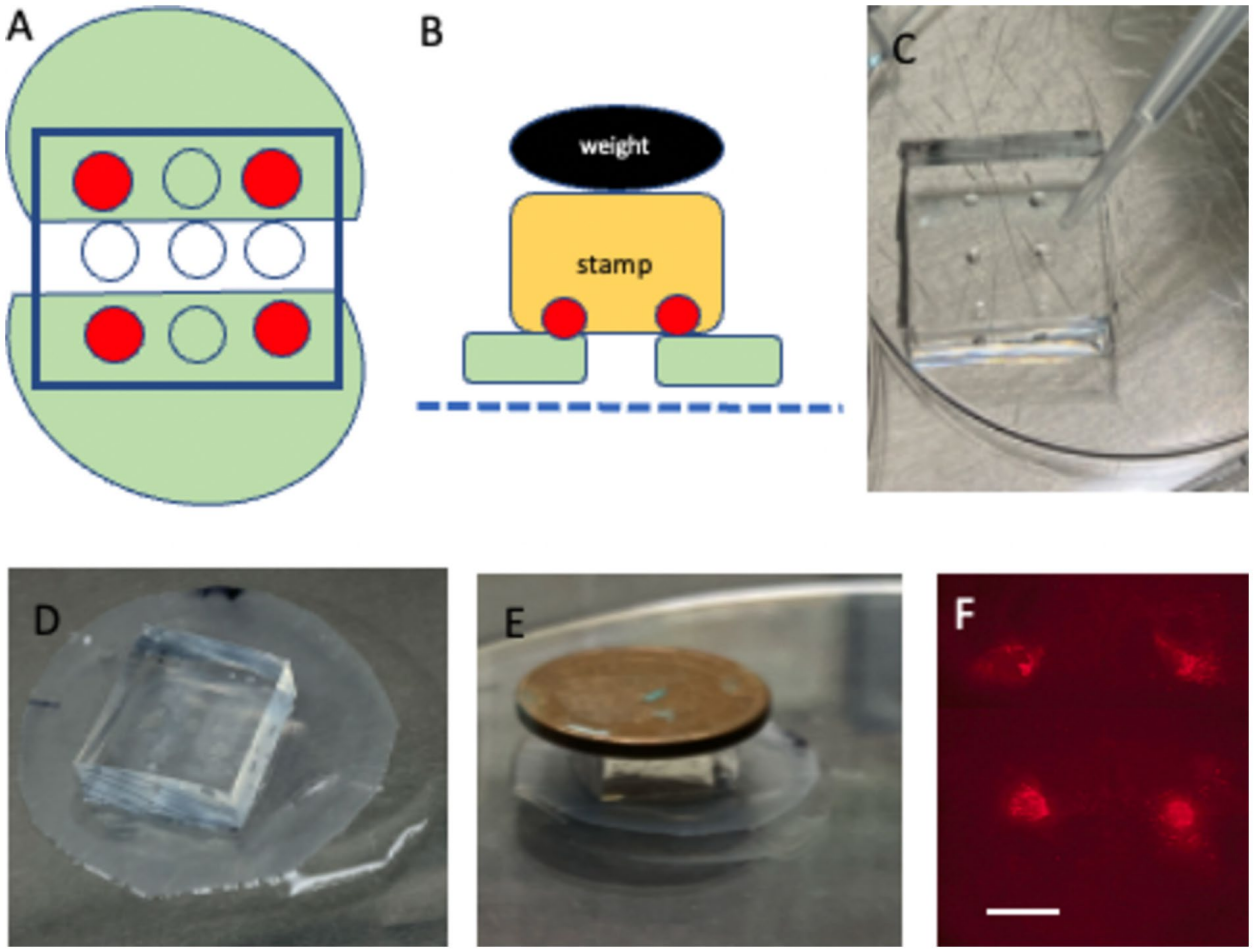
Microcontact printing directly onto brain slices. **(A)** Depicts two half-brain slices (green) positioned ~2 mm apart, with four collagen-stamped dots (red) placed on each slice. **(B)** Schematic showing how the stamp (yellow) is aligned and pressed onto the brain slices (green), situated on a semipermeable membrane (blue dotted line), using a 4 g weight (black). **(C)** Shows a close-up of the stamp loaded with collagen on four distinct dots. **(D)** Depicts the placement of the stamp directly above the two half-brain slices on the membrane. **(E)** Demonstrates the incubation process, where the stamp is pressed onto the slices with 4 g weight for 15 min. **(F)** Shows fluorescence imaging of the four microcontact prints on the brain slice. Please note that the stamp in **(D)** covers the whole-brain slices. Scale bars: C = 4 mm, D = 4.7 mm, E = 8 mm, and F = 906 μm.

- Conduct all steps under laminar flow conditions.
- Place the sterilized PDMS stamp in a 6-well plate with the patterned side (3×3 dots) facing upward.
- Prepare a collagen mixture on ice by sequentially adding the following components into an Eppendorf tube:

- 66.7 μL Bovine collagen solution type I (3 mg/mL, Sigma, 804,592).
- +10 μL 10x phosphate-buffered saline (PBS).
- +0.8 μL 1 N NaOH.
- +2.5 μL laminin antibody (Sigma-Aldrich, L9393), GFAP antibody (Merck Millipore AB5541), or 1x PBS (control).
- +7.5 μL 1x PBS.
- Vortex the mixture briefly, spin it down, and keep the solution on ice.
- Carefully apply ~0.5 μL of the collagen solution onto each 400-μm dot under a microscope (Figures 3A–C).
- Incubate the stamps at 37°C in a sterile chamber.
- Prepare a fresh stock PEG solution (2.5 mg 4arm-PEG succinimidyl succinate in 200 μL PBS, Sigma-Aldrich, JKA7006), vortex, and store it on ice until use.

### Microcontact printing onto brain slices

- Under sterile laminar flow conditions, remove the Biopore membrane containing the half-brain slices from the insert and place it in a Petri dish with the slices facing upward.
- Confirm slice orientation (hippocampus should be dorsal) and mark the position if necessary with a black fine liner.
- Apply 100 μL of PEG solution beneath the Biopore membrane containing the slices.
- Align the collagen-loaded PDMS stamp over the slices, ensuring each half-brain slice is covered with two dots (upper left and right and lower left and right) (Figure 3D).
- Place a 4 g weight on top of the stamp and incubate the assembly at 37°C for 15 min in a sterile chamber (Figures 3B,E).
- Carefully remove the weight and detach the stamp from the slices.
- Transfer the Biopore membrane with the slices back to its original membrane and insert it in the 6-well plate for further incubation.
- Check the dots under the microscope after labeling (Figure 3F).

### Fluorescent labeling of laminin and GFAP

- Replace the culture medium with 1 mL of fresh slice medium containing fluorescent secondary antibodies.

- Green anti-rabbit-Alexa-488 (laminin, 1:400; Invitrogen, 21206).
- Green anti-chicken-Alexa-488 (GFAP, 1:400).
- Alternatively, red Alexa-546 for either target.
- Incubate the slices overnight at 37°C.
- The following day, wash the slices by replacing the medium and capturing the first fluorescence images under a microscope.

### Live-cell imaging

- Under laminar flow, transfer the Biopore membrane with the slices to a 6-well plate containing 100 μL of sterile PBS, ensuring the slices face downward.
- Use an inverse fluorescence microscope to locate the fluorescently labeled dots, focusing on the dorsal hippocampal region.
- Capture images at 2x, 4x, or 10x magnification using a Bresser MicroCam pro HDMI camera with an exposure time of 120 ms and a gain of 30.
- After imaging, transfer the slices back to the original membrane insert for continued incubation, enabling long-term imaging for up to 100 days.

### Stimulate slices

One day after microcontact printing and labeling of the slices (2 weeks in culture), the slices were stimulated with varying concentrations of fibroblast growth factor-2 (FGF-2) or lipopolysaccharide (LPS). For low-dose conditions, the slices were incubated with 10 ng/mL FGF-2 or 100 ng/mL LPS for up to 100 days. High-dose conditions involved incubation with 1 μg/mL FGF-2 or 10 μg/mL LPS for 7 days. Negative controls were treated with PBS under identical conditions.

### Propidium iodide and DAPI staining

To analyze cell viability, 100-day-old cultured slices were incubated in 2 μg/mL PI while still living. The slices were then fixed with 4% paraformaldehyde for 60 min, washed, and counterstained with blue fluorescent nuclear DAPI for an additional 60 min. Following the staining protocol, the slices were washed and analyzed under fluorescence microscopy to assess cell death.

### Microscopic evaluation

Microscopic analysis (Figures 2F,G, 3F) was performed using a Leica DM *IRB* inverse fluorescence microscope with long-working-distance objectives. Green fluorescence (Alexa-488) was visualized using the L5 filter (excitation: 480/40; cutoff: 505; emission: 527/30), while red fluorescence (Alexa-546) was visualized using the Y3 filter (excitation: 535/50; cutoff: 565; emission: 610/75).

## Results, discussion, and perspectives

The present study introduces a novel methodology to fluorescently label astrocytes or vessels in organotypic brain slices, enabling real-time visualization of their reorganization. This approach integrates the preparation of organotypic mouse brain slices from postnatal day 8–10 mice, microcontact printing of laminin or GFAP antibodies, fluorescent secondary antibody labeling, and live-cell imaging for up to 100 days using a conventional inverse fluorescence microscope.

### Organotypic brain slices

Organotypic brain slices have been an integral part of our laboratory’s work for nearly 25 years, enabling the culture of various brain regions such as the nucleus basalis of Meynert, cortex, striatum, and ventral mesencephalon, prepared as coronal or sagittal slices (review Humpel, 2015). Brain slices are usually harvested from postnatal days 8–10 in mice as they consistently yield the highest survival rates, whereas culturing adult mouse brain slices remains challenging (Humpel, 2015). The slices, cultured at a thickness of 150 μm, typically adhere well to the membrane and flatten to transparency—a key indicator of slice viability and quality. Slices that fail to flatten (~10%) are excluded from experiments.

In this study, two half-brain slices were positioned at the hippocampal level with a 2-mm gap. This setup provided several benefits: clear orientation of hippocampal regions, reproducible sampling from two different mice (*n* = 2), a direct comparison between antibody-loaded and PBS control conditions on opposite sides, and rapid localization of the four labeled dots under fluorescence microscopy. This precise alignment minimizes light exposure, reducing the risk of fluorescence signal degradation.

### Microcontact printing on slices

Culturing organotypic brain slices requires substantial technical expertise, and microcontact printing adds additional complexity. PDMS stamps, fabricated from commercially available master plates (GeSiM), are easily customizable for various experimental designs.

For this study, a 3×3 dot master plate with 400-μm-diameter dots was selected after preliminary testing of 200–1,000-μm dots, with 400 μm providing the most consistent results. This design balances experimental versatility with cost-efficiency. The collagen antibody solution, loaded onto the 400-μm dots, demands precision due to the small volume applied (0.5 μL). Weight application (4 g) during printing was optimal for preserving slice integrity. Laminin and GFAP antibodies were effectively labeled extracellular antigens, while other antibodies requiring intracellular access were less successful, underscoring the need for application-specific optimization.

### Live-cell imaging of laminin+ vessel

**Figure 4:**
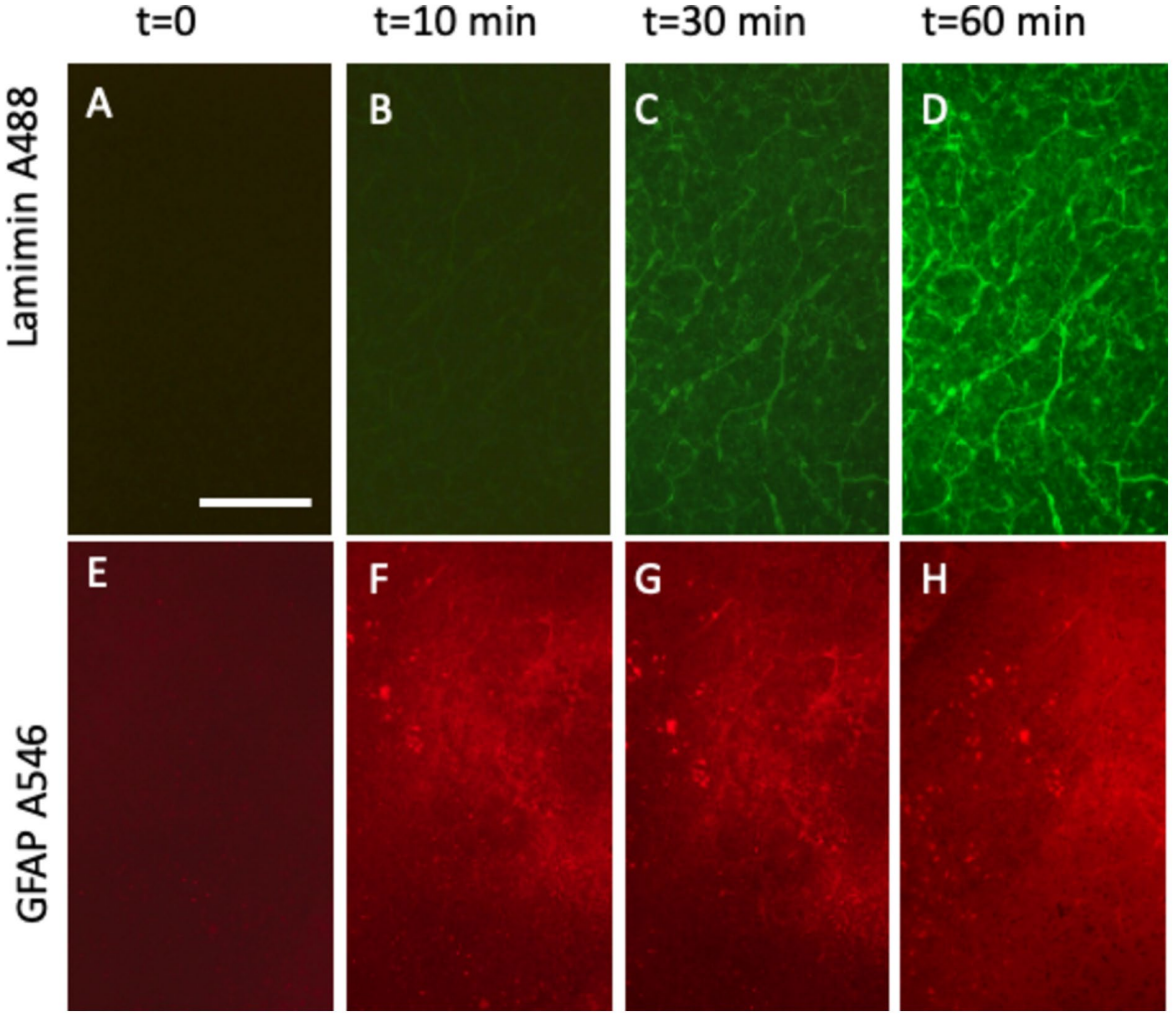
Time-dependent detection of laminin+ vessel **(A–D)** and astroglial GFAP **(E–H)** visualized using fluorescence inverse microscopy. Following microcontact printing, secondary antibodies—anti-rabbit Alexa-488 for laminin **(A–D)** and anti-chicken Alexa-546 **(E–H)**—were applied, and fluorescence signals were captured at 0 **(A,E)**, 10 min **(B,F)**, 30 min **(C,G)**, and 60 min **(D,H)**. Please note that **(A–D)** (vessel) show the same slice area, visualized at four different time points. **(E–H)** (Astroglia) also show the same slice area, visualized at four time points. Scale bars: A = 270 μm (all panels).

**Figure 5:**
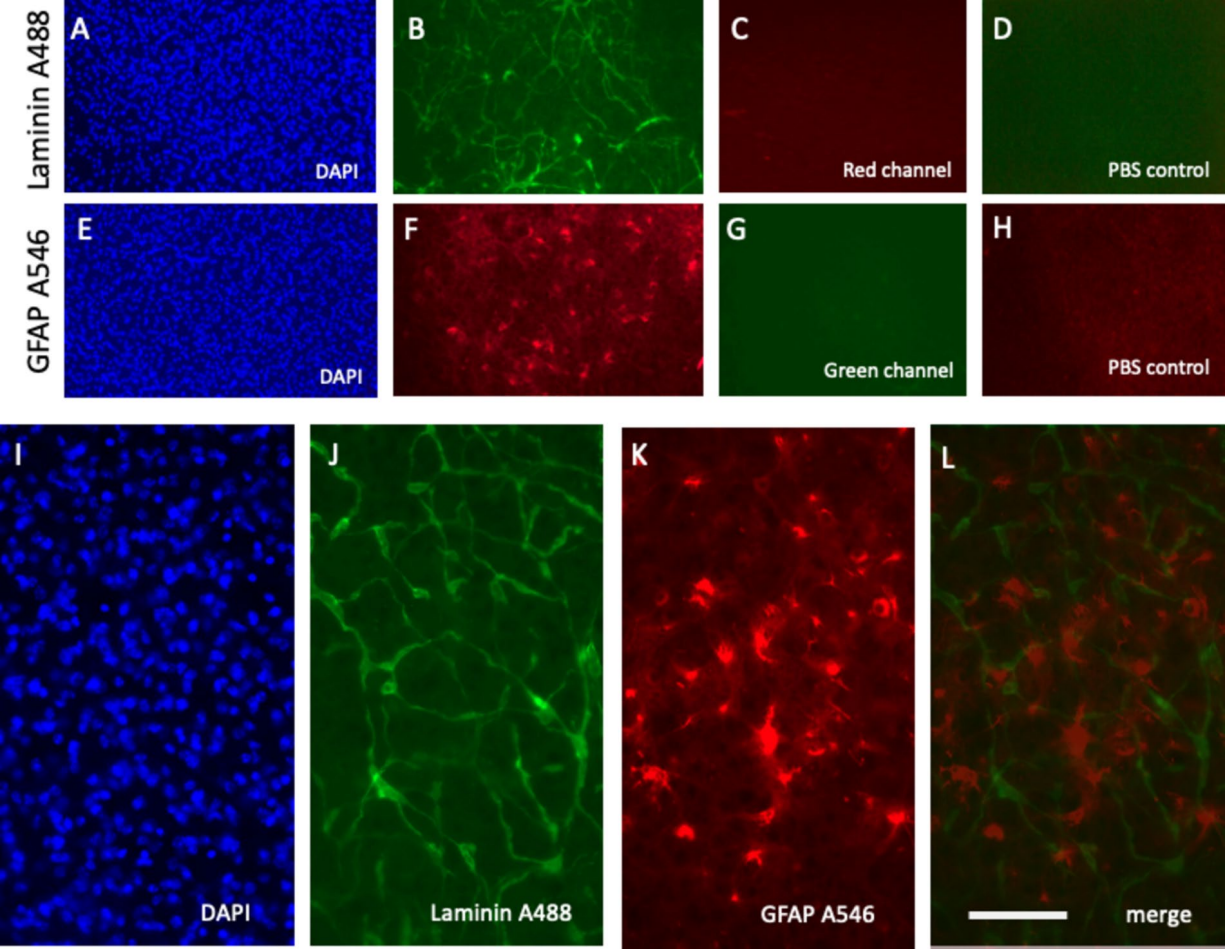
Specific detection of laminin+ vessel and glial fibrillary acid protein (GFAP)+ astroglia microcontact printed onto brain slices alongside respective negative controls. Laminin was microcontact-printed and visualized using a green anti-rabbit Alexa-488 antibody **(B)**, with blue nuclear DAPI as a counterstain **(A)**. Notably, no signal was observed in the red channel **(C)**. Similarly, GFAP was microcontact-printed and visualized using a red anti-chicken Alexa-546 antibody **(F)**, counterstained with blue fluorescent nuclear DAPI **(E)**. The absence of green channel signals confirms specificity **(G)**. As an additional negative control, phosphate-buffered saline (PBS) was microcontact-printed, showing only background signals in the green **(D)** and red **(H)** channels. Higher magnification images highlight the laminin Alexa-488 signal (green, **J**) and GFAP Alexa-546 signal (red, **K**), merged in **(L)** and counterstained with DAPI **(I)**. Scale bars: L = 270 μm **(A–H)** and 108 μm **(I–L)**.

Laminin, a well-established marker for blood vessels, has been widely documented in previous studies (Grant and Kleinman, 1997; Schnaper et al., 1993; Kosmehl et al., 1996; Engvall, 1993). Our laboratory has extensive experience analyzing brain vessels in organotypic brain slices using laminin staining (Moser et al., 2003; Ucar et al., 2021; Hutter-Schmid et al., 2015). In this study, laminin was labeled effectively using an anti-rabbit A488 green fluorescent antibody, yielding high-intensity signals within 30–60 min of incubation (Figures 4A–D; Figure 5B). DAPI staining confirmed the integrity of the nuclei with no observed cellular damage (Figure 5A). The specificity of the signal was validated by the absence of fluorescence in the red channel (Figure 5C) and PBS-treated control slices (Figure 5D).

### Live-cell imaging of GFAP+ astroglia

GFAP, a well-established marker for reactive astrocytes (Eng, 1985; Eng and Ghirnikar, 1994; Brenner, 2014; Jurga et al., 2021), has been extensively studied and is particularly valuable when stained near beta-amyloid plaques (Daschil and Humpel, 2016). In this study, GFAP antibodies effectively labeled astroglia and were visualized using anti-chicken Alexa-546 red fluorescent antibodies (Figure 5F). Slices were incubated directly in the medium, and a robust signal was observed as early as 10 min after incubation (Figures 4E–H). No damage to DAPI+ cells was noted (Figure 5E). The specificity of the signal was validated as no fluorescence was detected in the green channel (Figure 5G) or when slices were treated with a negative PBS control (Figure 5H).

### Co-staining of laminin+ vessels and GFAP+ astroglia

A notable advantage of this methodology is the simultaneous labeling of vessels and astroglia. Slices are microcontact-printed with laminin and GFAP antibodies and subsequently incubated with the anti-rabbit Alexa-488 (green) and anti-chicken Alexa-546 (red) antibodies, respectively. High-magnification images revealed co-staining of laminin+ vessels (green, Figure 5J) and GFAP+ astroglia (red, Figure 5K), with merged images showing clear co-localization (Figure 5L). Nuclear DAPI staining confirmed cell viability (Figure 5I).

### Live-cell imaging of laminin+ vessels for up to 20 days

**Figure 6:**
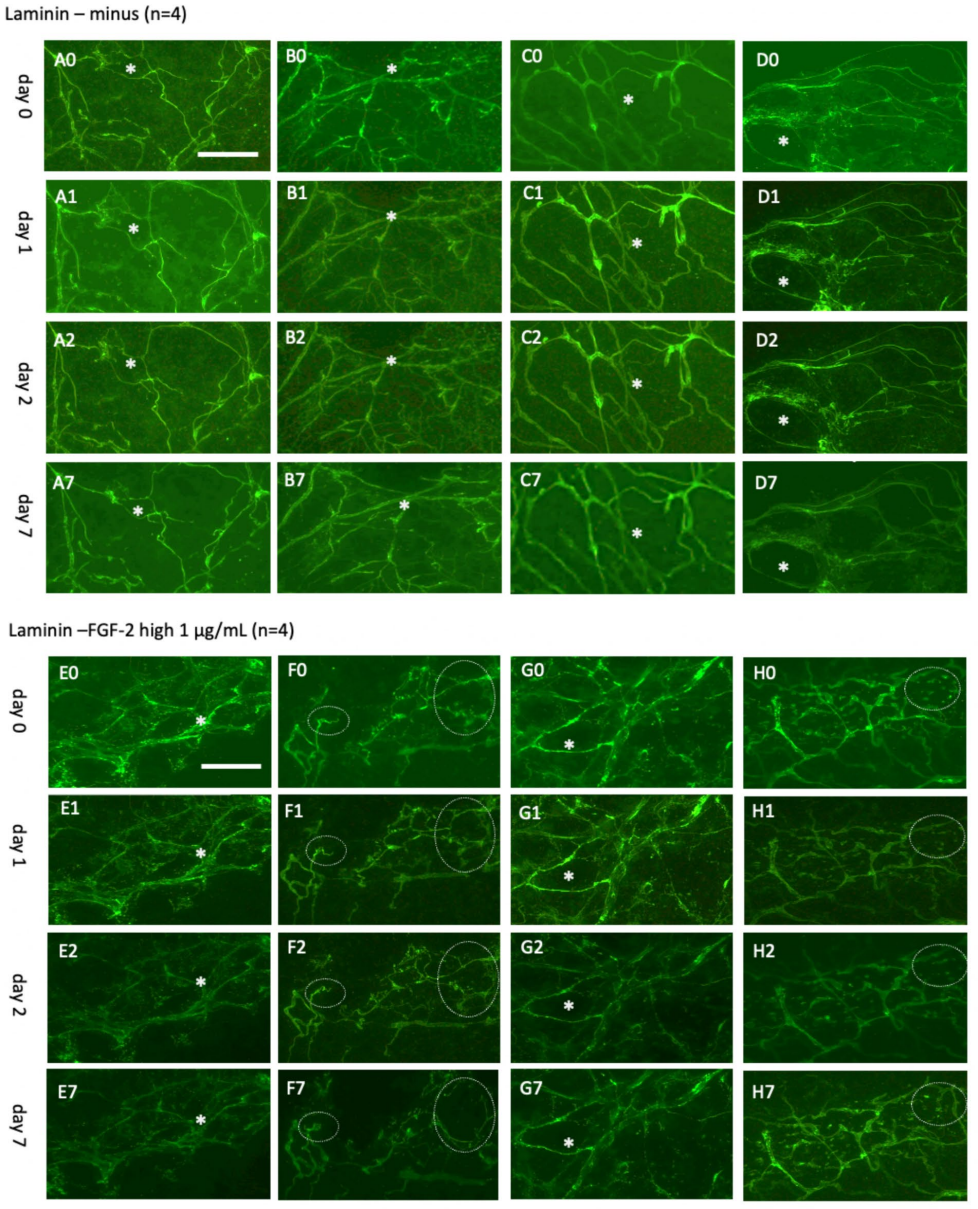
Laminin+ vessels incubated either without **(A–D)** or with a high concentration of fibroblast growth factor-2 (FGF-2, 1 μg/mL) **(E–H)** for 0 days **(A0–H0)**, 1 day **(A1–H1)**, 2 days **(A2–H2)**, or 7 days **(A7–H7)**. Organotypic brain slices were microcontact-printed with laminin to label vessels and photographed at the specified time points. White stars (*) mark identical regions within corresponding slices, and white dotted circles highlight areas where significant changes were observed. Laminin+ vessels maintained their network structure in a normal slice media, showing no significant changes. However, a clear tendency for laminin+ reorganization was observed in at least two out of four slices **(F7,H7)**. Specifically, **(F7)** depicts the formation of an expanded laminin+ area, potentially representing a vessel-like region. Scale bars: A0 and E0 = 108 μm (all panels).

**Figure 7:**
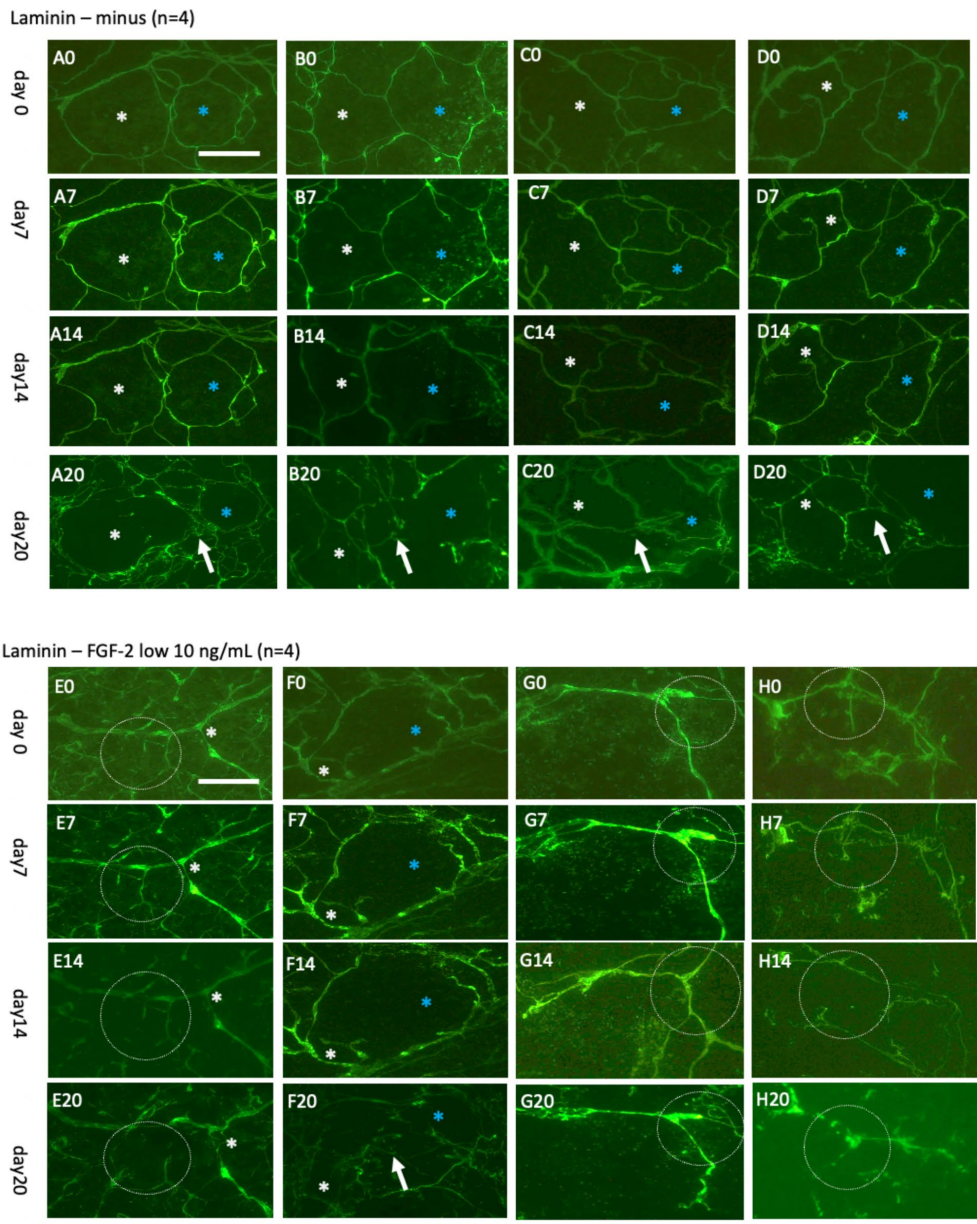
Laminin+ vessels incubated either without **(A–D)** or with a low concentration of fibroblast growth factor-2 (FGF-2, 10 ng/mL) **(E–H)** for 0 **(A0–H0)**, 7 days **(A7–H7)**, 14 days **(A14–H14)**, or 20 days **(A20–H20)**. Organotypic brain slices were microcontact-printed with laminin to label vessels and photographed at the specified intervals. White and blue stars (*) highlight the same area in respective slices, while white dotted circles and arrows indicate significant changes. Laminin+ vessels exhibited no significant alterations in normal medium for up to 14 days but demonstrated a pronounced reorganization into larger laminin+ by 20 days **(A20–D20)**. Slices treated with FGF-2 displayed distinct modifications in the laminin+ network as early as day 7 **(E7–H7)**, becoming more prominent by day 14 **(E14–H14)** and, subsequently, day 20 **(E20–H20)**. Scale bars: A0 and E0 = 108 μm (all panels).

Initial investigations tracked laminin+ vessels for up to 7 days (Figure 6) and 20 days (Figure 7). The results demonstrated the stability of the Alexa-488 fluorescent dye for up to 20 days, enabling effective monitoring of laminin+ vessels. Interestingly, while the vessel network showed little change during the initial period (Figures 6A–D), it self-reorganized into larger “vessel-like” structures after 20 days (Figures 7A–D). This stability and reorganization were validated by observing the same vessel across time points, confirming the specificity and reproducibility of the findings.

To investigate vessel activation, organotypic brain slices were incubated with FGF-2, a growth factor extensively documented for its potent pro-angiogenic properties and its ability to drive vascular remodeling (Schweigerer, 1989; Vlodavsky et al., 1991; Rusnati and Presta, 1996; Chen et al., 2004; Presta et al., 2005; Murakami and Simons, 2008). These findings are consistent with observations from our laboratory (Ucar et al., 2020). Acute exposure to a high dose of FGF-2 (1 μg/mL; Figures 6E–H) resulted in discernible vessel pattern alterations within 7 days, indicating rapid and robust vascular response (Figure 6H). Chronic exposure to a low concentration (10 ng/mL; Figures 7E–H) demonstrated progressive and sustained effects, with marked changes in vessel morphology observed as early as 7 days and further intensifying by 20 days (Figure 6). These results underscore the dose-dependent efficacy of FGF-2 in promoting vascular dynamics in an organotypic brain slice model.

### Live-cell imaging of GFAP+ astroglia up to 20 days

**Figure 8:**
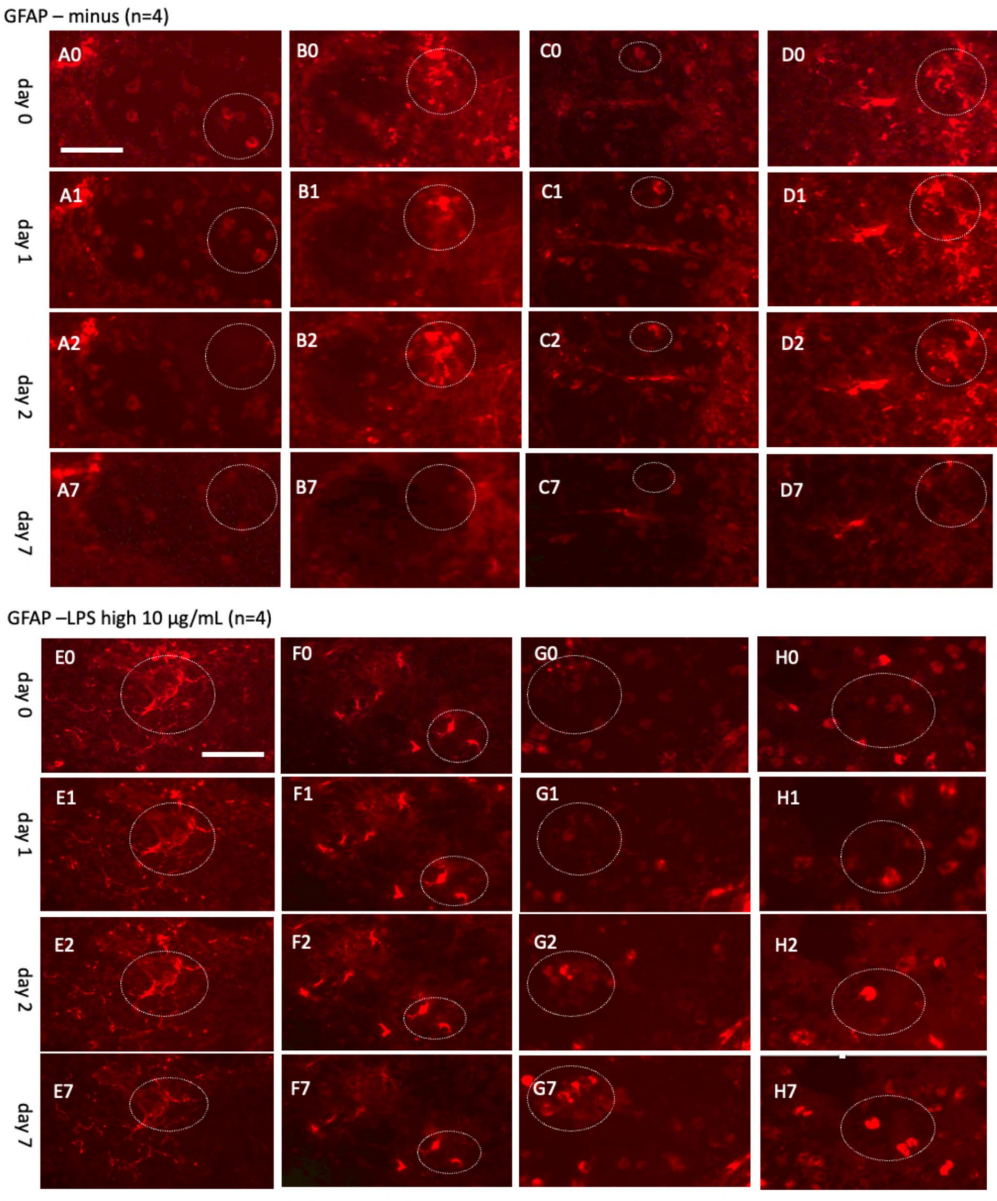
Glial fibrillary acidic protein (GFAP)+ astrocytes incubated either without **(A–D)** or with a high concentration of lipopolysaccharide (LPS, 10 μg/mL) **(E–H)** for 0 **(A0–H0)**, 1 day **(A1–H1)**, 2 days **(A2–H2)**, or 7 days **(A7–H7)**. Organotypic brain slices were microcontact-printed with GFAP to label astroglia and photographed at specified intervals. White dotted circles denote areas where notable changes were observed. GFAP+ astroglial density markedly decreased in slices incubated in normal media, whereas LPS-treated slices exhibited a substantial increase in GFAP+ astroglia by day 7 **(E7–H7)**. Scale bars: A0 and E0 = 108 μm (all panels).

**Figure 9:**
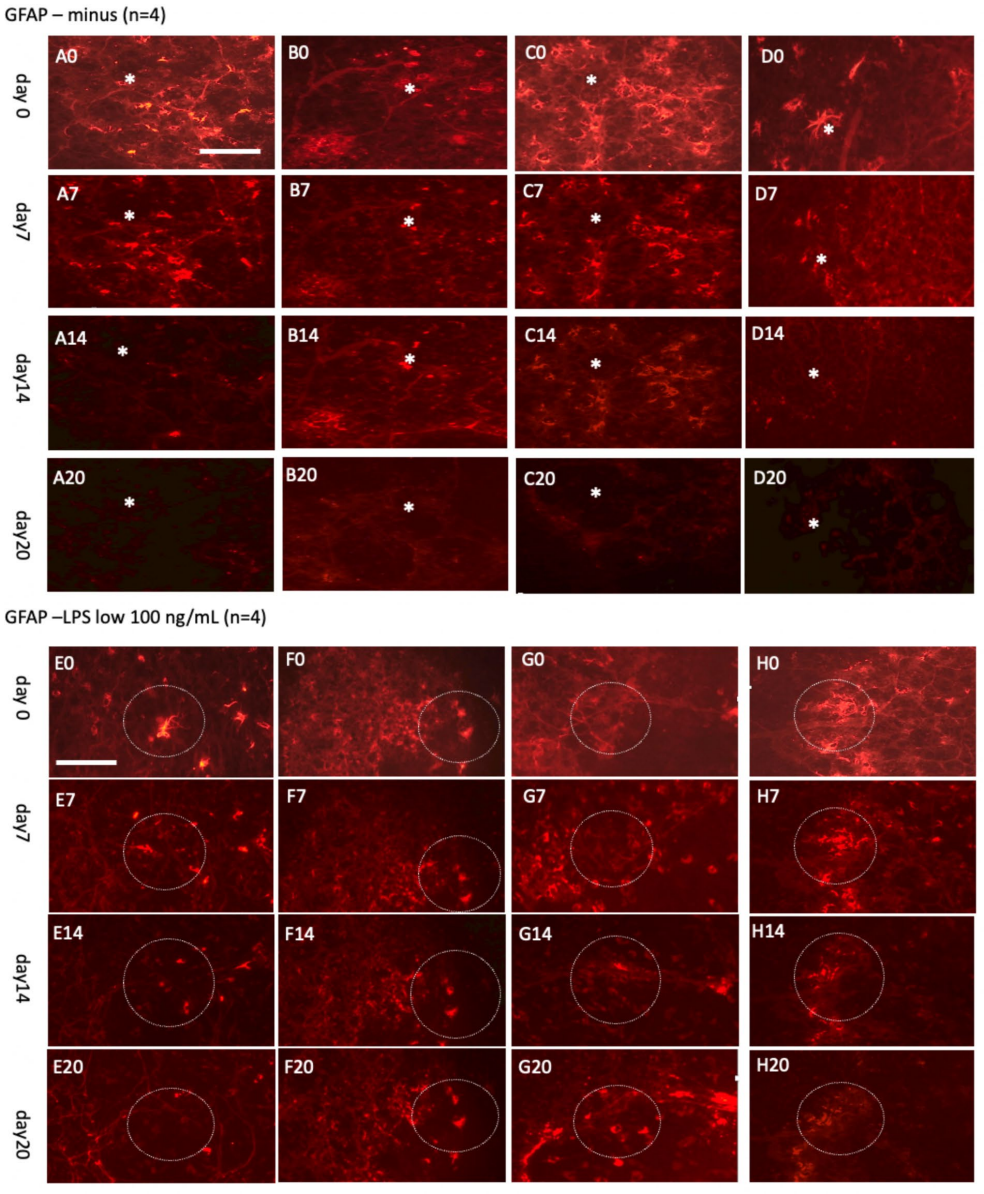
Glial fibrillary acidic protein (GFAP)+ astrocytes incubated either without **(A–D)** or with a low concentration of lipopolysaccharide (LPS, 100 ng/mL) **(E–H)** for 0 days **(A0–H0)**, 7 days **(A7–H7)**, 14 days **(A14–H14)**, or 20 days **(A20–H20)**. Organotypic brain slices were microcontact-printed with GFAP to label astroglia and photographed at the specified time points. White stars (*) highlight areas within the same slice that showed no observable changes, while white dotted circles indicate regions with noticeable alterations. The density of GFAP+ astroglia significantly decreased in slices incubated in normal media for up to 20 days, whereas a marked enhancement in GFAP+ astroglia was observed when incubated with LPS **(E20–H20)**. Scale bars: A0 and E0 = 108 μm (all panels).

In parallel, astroglia and vessel were investigated over 7 days (Figure 8) and extended up to 20 days (Figure 9). The analysis involved co-staining with Alexa-488 for green vessels and Alexa-546 for red astrocytes, enabling straightforward switching between fluorescence channels. The findings demonstrated that the Alexa-546 dye remains stable for up to 20 days, allowing consistent tracking of GFAP+ astrocytes during this period. Strong GFAP+ astrocyte staining was observed on days 0 and 7 (Figure 8). However, by day 14, the signal intensity had significantly diminished and partially faded by day 20 (Figure 9).

To explore astroglial activation, the slices were exposed to LPS, a well-established inflammatory stimulus known to induce reactive astrogliosis through its interaction with toll-like receptor 4 (Hua et al., 2019; Hwang et al., 2016; Wang et al., 2010; da Silva et al., 2023). This approach aligns with our laboratory’s previous methodologies (Weis and Humpel, 2002). Acute treatment with a high dose of LPS (10 μg/mL; Figures 8E–H) elicited strong GFAP+ astroglial activation by 7 days, evidenced by increased GFAP immunoreactivity. Chronic exposure to a low dose of LPS (100 ng/mL; Figures 9E–H) sustained robust GFAP staining, with astrocytic activation persisting for up to 20 days. Notably, GFAP staining diminished when slices were cultured without LPS, suggesting that unstimulated astrocytes may downregulate GFAP expression or release it into the medium over time. In contrast, LPS-activated astroglia appeared to stabilize GFAP expression, maintaining their reactive state or potentially reactivating under continuous stimulation.

### Live-cell imaging of vessel and astroglia for 50 days

**Figure 10:**
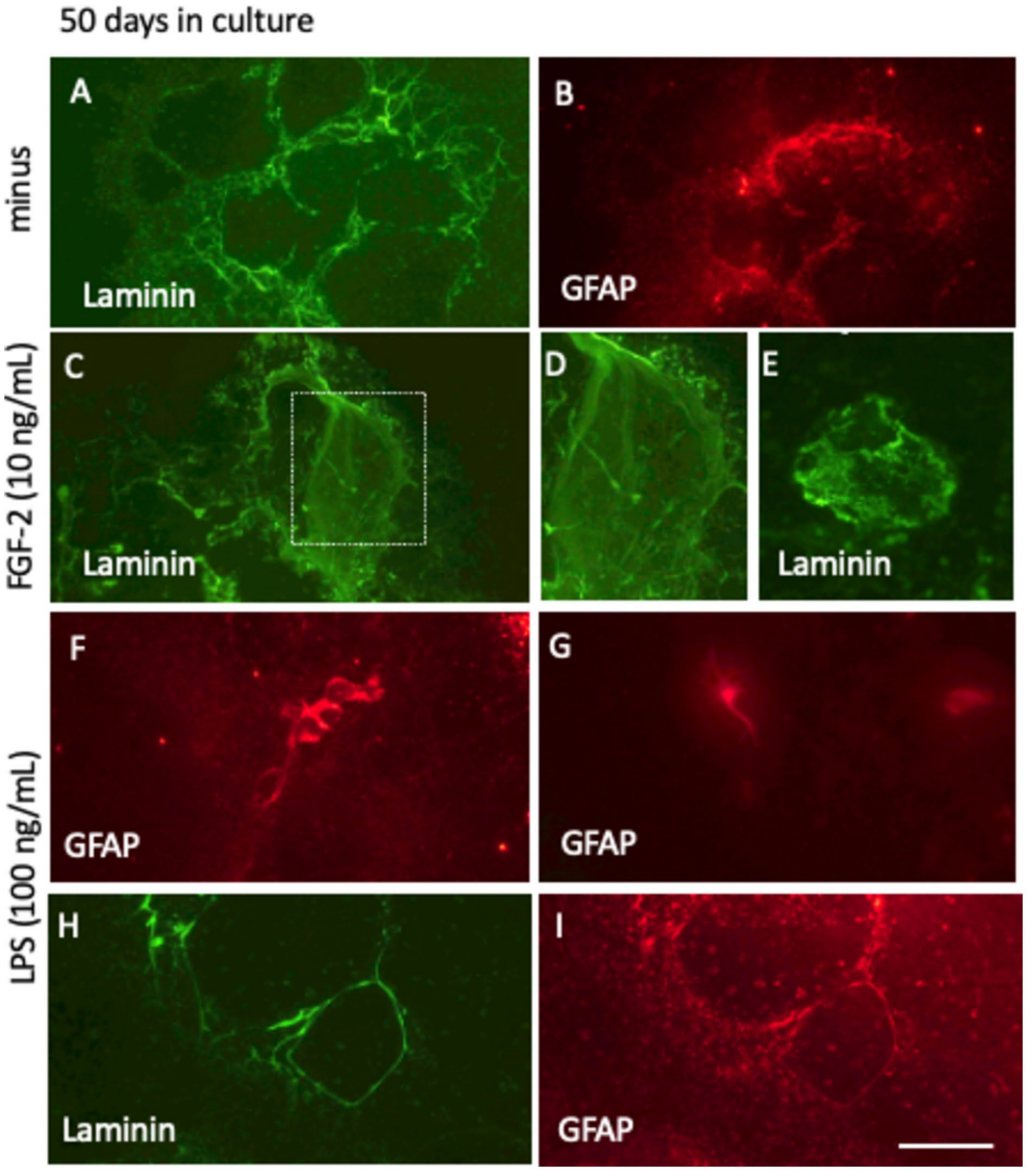
Long-term culture (50 days) of laminin+ vessel **(A,C–E,H)** and astroglial GFAP **(B,F,G,I)** visualized through fluorescence inverse microscopy. After microcontact printing, secondary antibodies—anti-rabbit Alexa-488 for laminin and anti-chicken Alexa 546 for GFAP—were applied, followed by incubation for 50 days at 37°C and 5% CO_2_. Conditions included fibroblast growth factor-2 (FGF-2, 10 ng/mL; **C–E**) or lipopolysaccharide (LPS, 100 ng/mL; **F–I**). Laminin+ vessels exhibited a well-organized vessel-like network (A, H), with FGF-2 treatment promoting a pronounced clumping pattern **(C–E)**. Astroglial GFAP+ cells either surrounded the vessels **(B,I)** or formed dense clusters and ramified structures **(F,G)**. Scale bars: I = 105 μm (all panels).

After 50 days in culture without FGF-2 supplementation, the laminin+ network developed substantially, forming significantly more vessel-like structures (Figure 10A). Notably, portions of the laminin+ network were partially covered by the GFAP+ astrocytes, indicating interactions between astrocytic and vascular components (Figure 10B). In slices treated with 10 ng/mL FGF-2 for 50 days, a diffuse, clot-like laminin+ pattern emerged in some, though not all, samples, suggesting a heterogeneous response to growth factor stimulation (Figures 10C–E). When cultured with a low dose of LPS (100 ng/mL) over the same period, the laminin+ network co-localized with GFAP+ astrocytic structures. In addition, distinct GFAP+ clusters (Figure 10F) and ramified astrocytes (Figure 10G) were observed. LPS treatment further enhanced the laminin and GFAP+ networks, forming large, single vessel-like compartments and highlighting a robust structural reorganization (Figures 10H,I).

### Live cell imaging of vessel and astroglia for 100 days

**Figure 11:**
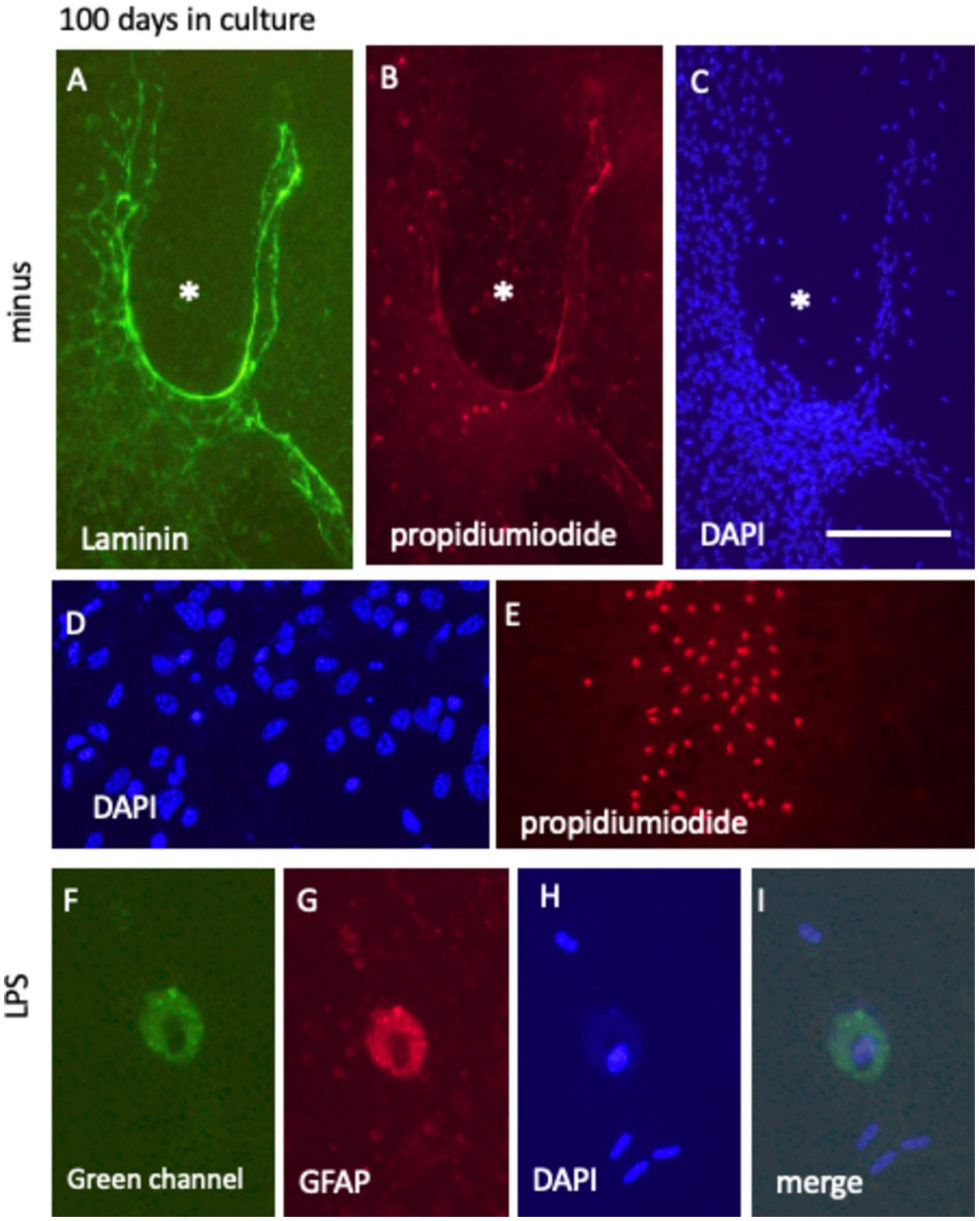
Long-term culture (100 days) of laminin+ vessel **(A)** and astroglial GFAP **(D)**, counterstained with blue fluorescent nuclear DAPI **(C,D,H)** and visualized using the cell death-specific red fluorescent nuclear propidium iodide (PI) **(B,E,G)**. **(A–C)** Show a characteristic vessel-like region surrounded by cell-free areas (denoted by * in **C**), with minimal PI+ cell death evident in **(B)**. **(D,E)** Highlight PI+ nuclei predominantly located at the edges of the brain slice. In contrast, DAPI+ nuclei in **(D)** indicate cellular survival within the slice after 100 days in culture. **(F–I)** Depict GFAP+ cells post-LPS treatment, characterized by the absence of PI+ nuclei, with nuclei remaining DAPI+ and intact. Notably, these cells exhibit pronounced green autofluorescence **(F)**. Scale bars: C = 105 μm (applicable to **A–C,E**), D = 40 μm, and 25 F = 25 μm (applicable to **F–I**).

After 100 days in culture media, with or without 10 ng/mL FGF-2 supplementation, the laminin+ vessel-like network exhibited further maturation. DAPI staining revealed that the vessel-like formations lacked cells within their structure, as indicated by the absence of DAPI+ nuclei in these regions (highlighted by white stars in Figures 11A–C). Surrounding these formations, abundant DAPI+ nuclei suggested that the overall viability of the brain slices was preserved, even after prolonged culture (Figures 11B,D). However, in some isolated areas, particularly at the periphery of the slices, strong PI+ nuclei were detected, indicative of localized cell death (Figure 11E).

When the slices were incubated in culture media supplemented with a low dose of 100 ng/mL LPS for 100 days, prominent “macrophage-like” cells were frequently observed. These cells exhibited red fluorescence, potentially due to autofluorescence, and concurrently displayed strong green fluorescent autofluorescence (Figures 11F,G). Despite these fluorescence patterns, the cells were confirmed viable, as evidenced by the absence of PI+ nuclei, indicative of cell death, and the presence of DAPI+ nuclei, reflecting intact nuclear integrity (Figures 11H,I).

## Perspectives on live-cell imaging

This methodological perspective introduces a novel technique to label blood vessels and astroglia in brain slices for live-cell imaging, offering significant potential for application across diverse research laboratories. The method is straightforward yet requires proficiency in culturing organotypic brain slices and handling microcontact prints. A notable advantage is that transgenic mice expressing fluorescent reporter genes are unnecessary as the method can theoretically employ any antibody, protein, or molecule of interest. Through optimization, we have demonstrated the feasibility of directly printing antibodies onto brain slices to label specific cells. Our findings suggest that these antibodies likely target extracellular domains for effective staining.

We validated this approach using laminin and GFAP antibodies and anticipate its applicability with other antibodies, provided proper optimization is conducted. Our previous work has also shown success in printing proteins such as NGF and GDNF onto membranes, though applying this directly to brain slices may prove more challenging due to cellular uptake requirements. This limitation highlights the need to explore co-loading matrices, such as incorporating delivery materials (e.g., liposomes) into collagen matrices, to enhance protein uptake. With the ability to effectively label areas as small as 400 μm on brain slices, we are optimistic about the potential for even smaller regions to be labeled using advanced laser-assisted techniques. The method also allows for the simultaneous study of two cells via co-loading molecules, with ongoing efforts to extend this capability to microglia using alternative fluorescent dyes.

The proof-of-principle demonstrated in this study highlights the feasibility of live-cell imaging to track vascular and glial networks over an extended period. This method can visualize entire vascular networks over several weeks, easily identifying and monitoring the same regions. GFAP+ astroglia were also imaged at higher magnifications; however, precise identification of the same cells over time requires careful execution. The co-staining with the laminin network provided spatial orientation. Still, future enhancements could involve cell culture chambers coupled with fluorescence microscopy to maintain constant visualization of the microscopic field over days.

Our findings show that blood vessels within the slices can reorganize over time, influenced by intrinsic or external factors, such as growth factors (e.g., FGF-2). Reactive astroglia exhibited diminishing activity over time but were reactivated by exposure to LPS, underscoring the dynamic nature of the brain slice environment. While these qualitative observations were a key focus, quantitative measurements were not performed as they fell outside the scope of this study. Cell viability was confirmed through propidium iodide staining and the presence of healthy DAPI+ nuclei, indicating that the slices remain viable for up to 100 days. Ongoing work includes labeling neurons with receptor-specific antibodies, such as those targeting cholinergic or dopaminergic neurons, to observe nerve fiber growth. Furthermore, we aim to investigate microglial activity using CD-specific antigens (e.g., CD11b or CD11c) under inflammatory conditions, paving the way for comprehensive studies of cellular interactions within brain slices.

This technique offers significant advantages but is not without its limitations. First, the current printing field, measuring 400 μm, is relatively large and could benefit from refinement through commercial automated laser-assisted systems. Such systems could optimally facilitate printing hundreds of points, enabling physiological interactions within the slice and potentially creating a simplified brain-on-a-chip network. Alternatively, nanoprinting techniques could be explored to label individual neurons in brain slices and assess their development more precisely.

Second, while this study demonstrated proof-of-principle labeling of vessels and astroglia, positive labeling of other brain cells remains untested. Long-term cell survival was also not systematically evaluated. Although live staining with propidium iodide could provide insights, this approach is limited by its toxicity during prolonged incubation. Future investigations might consider labeling all cells with a nuclear dye, such as blue fluorescent DAPI, provided that its effects on cell viability are thoroughly assessed. In addition, fluorescent tracing dyes, including neurotracers or retrograde labeling dyes, could be used to expand the scope of this method.

Third, applying the stamps directly onto brain slices involves using a 4 g weight, which can exert mechanical stress and potentially damage the slices. To mitigate this, optimizing the thickness of the slices is critical. However, excessively thick slices may fail to flatten properly, complicating the procedure. Addressing these challenges will require methodological refinement and skilled handling by experienced technicians.

In conclusion, this perspective underscores the utility of live-cell imaging of laminin+ vessels and GFAP+ astroglia in mouse organotypic brain slices. Using a conventional inverse long-distance fluorescence microscope, the technique offers a straightforward and reliable means of tracking cellular dynamics for up to 100 days. Future research will extend this methodology to label microglia and neurons, potentially establishing a quadruplicate model incorporating additional fluorescent dyes (e.g., high red, blue, or far-red). Such advancements could enable the visualization of interactions among vessels, astroglia, neurons, and microglia, furthering our understanding of cellular interplay in brain slices through live-cell imaging.

## Data Availability

The original contributions presented in the study are included in the article/supplementary material, further inquiries can be directed to the corresponding author.
